# *Gracilaria chorda* attenuates obesity‐related muscle wasting through activation of SIRT1/PGC1α in skeletal muscle of mice

**DOI:** 10.1002/fsn3.4157

**Published:** 2024-04-04

**Authors:** Ahyoung Yoo, Jiyun Ahn, Hyo Deok Seo, Jeong‐Hoon Hahm, Min Jung Kim, Chang Hwa Jung, Tae Youl Ha

**Affiliations:** ^1^ Division of Food Functionality Research Korea Food Research Institute Wanju‐gun Korea; ^2^ Division of Food Biotechnology University of Science and Technology Daejeon Korea

**Keywords:** *Gracilaria chorda*, obesity‐related skeletal muscle wasting, SIRT1/PGC1α, skeletal muscle atrophy, skeletal muscle hypertrophy

## Abstract

*Gracilaria chorda* (GC) is a red algal species that is primarily consumed in Asia. Here, we investigated the effect of GC on obesity‐related skeletal muscle wasting. Furthermore, elucidating its impact on the activation of sirtuin 1 (SIRT1)/peroxisome proliferator‐activated receptor gamma coactivator 1α (PGC1α) constituted a critical aspect in understanding the underlying mechanism of action. In this study, 6‐week‐old male C57BL/6 mice were fed a high‐fat diet (HFD) for 8 weeks to induce obesity, then continued on the HFD for another 8 weeks while orally administered GC. GC decreased ectopic fat accumulation in skeletal muscle and increased muscle weight, size, and function in obese mice. Furthermore, GC reduced skeletal muscle atrophy and increased hypertrophy in mice. We hypothesized that the activation of SIRT1/PGC1α by GC regulates skeletal muscle atrophy and hypertrophy. We observed that GC increased the expression of SIRT1 and PGC1α in skeletal muscle of mice and in C2C12 cells, which increased mitochondrial function and biogenesis. In addition, when C2C12 cells were treated with the SIRT1‐specific inhibitor EX‐527, no changes were observed in the protein levels of SIRT1 and PGC1α in the GC‐treated C2C12 cells. Therefore, GC attenuated obesity‐related muscle wasting by improving mitochondrial function and biogenesis through the activation of SIRT1/PGC1α in the skeletal muscle of mice.

## Graphical abstract

GC increased skeletal muscle weight and improved muscle function in obese mice. GC reduced skeletal muscle atrophy and increased hypertrophy in mice. GC increased the expression of SIRT1 and PGC1α in vivo and in vitro, which increased mitochondrial function and biogenesis.

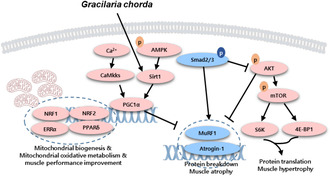

## 1 INTRODUCTION

Obesity is a disease in which abnormally large amounts of fat accumulate in the body and is accompanied by various metabolic diseases. Recently, the elderly have encountered a new obesity classification known as sarcopenia, which encompasses reduced skeletal muscle mass or strength alongside heightened obesity levels (Batsis & Villareal, 2018). Fat accumulation that occurs with age increases and is redistributed to harmful ectopic locations such as skeletal muscles (Biltz et al., 2020; Poggiogalle et al., 2022). Previous studies have demonstrated that elevated ectopic fat accumulation within the skeletal muscle adversely impacts protein synthesis and induces insulin resistance (Park et al., 2022; Ryan & Li, 2021). Furthermore, previous studies have reported that the accrual of adipose tissue amidst skeletal muscle tissue, as observed in aging and obesity, is associated with an accelerated progression of muscle atrophy (Zhu et al., 2019).

The accumulation of lipids in skeletal muscle leads to impaired mitochondrial function and upregulation of pro‐inflammatory cytokines secretion, consequently activating cellular stress signaling and leading to subsequent muscle atrophy (Rubio‐Ruiz et al., 2019). The compromised mitochondrial function additionally influences the expression of genes implicated in oxidative phosphorylation, modulated by peroxisome proliferator‐activated receptor (PPAR) gamma coactivator 1 alpha (PGC1α) regulation. Consequently, PGC1α plays a central role as the master regulator of mitochondrial biogenesis, wherein its activity is modulated through phosphorylation by AMP‐activated protein kinase (AMPK) and subsequent deacetylation by sirtuin 1 (SIRT1) (Devarshi et al., 2017). In addition, SIRT1 deacetylation of PGC1α has a suppressive effect on fat accumulation by modulating genes associated with mitochondrial and lipid metabolism (Finck & Kelly, 2006). Therefore, activation of the SIRT1‐PGC1 axis may be a major regulatory protein in obesity‐related muscle wasting.

*Gracilaria chorda* (GC) is a red alga, a type of seaweed, known to thrive in the coastal regions of several countries, such as Korea, Japan, and Sakhalin Island of Russia. It is widely used as a food ingredient because it is rich in fiber, minerals, and vitamins and is used for agar production (Armisen, 1995). It is known to have effects such as excretion of heavy metals through the smooth activity of the intestine and suppression of cholesterol deposition in blood vessels (Hong et al., 2016). Previous studies also demonstrated that GC has anticancer, anti‐inflammatory, antioxidative, antiadipogenic, insulin‐sensitive, neuritogenic and neuroprotective effects (Dang et al., 2008; Faten & Emad, 2009; Hong et al., 2016; Mohibbullah et al., 2015, 2016, 2018; Woo et al., 2013). In addition, sea grapes, which are a type of seaweed, have been shown to possess antihyperglycemic, antihypercholesterolemic, oxidative stress‐reducing, metabolic disorders‐addressing, and cardiometabolic syndrome‐addressing effects (Kuswari et al., 2021; Manoppo et al., 2022; Nurkolis et al., 2023). However, the precise effects of GC on obesity‐induced skeletal muscle wasting in skeletal muscle have yet to be fully elucidated. Therefore, this investigation aimed to assess whether GC protects against obesity‐related muscle wasting in C57BL/6 mice. Furthermore, our study aimed to examine the influence of GC on obesity‐induced mitochondrial dysfunction by assessing the activation of SIRT1/PGC1α in the skeletal muscle of mice.

## 2 MATERIALS AND METHODS

### 2.1 Sample preparation

GC was obtained from Samil Food Co. Ltd. (Gyeonggi‐do, Korea). The dried GC (0.5 kg) was subjected to extraction using 10 L of 50% ethanol at 80°C for a duration of 3 h. The obtained extract was subjected to filtration utilizing No. 2 filter paper (Toyo Roshi Kaisha, Tokyo, Japan) to remove solid matter. Subsequent to this, the extract was concentrated at 37°C under vacuum conditions to reduce solvent content, and freeze‐drying was employed to attain a desiccated state. The extraction yield was 0.95 g/100 g GC.

### 2.2 Ultra‐high performance liquid chromatography (UPLC)‐quadrupole time‐of‐flight (QTOF)‐mass spectrometry (MS) analysis

To perform the sample analysis, a coupling of an Acquity UPLC system (Waters Corporation, Milford, MA, USA) with a Waters SYNAPT G2‐Si mass spectrometer (Waters Corporation) was employed. Using an Acquity UPLC BEH C18 column (2.1 × 100 mm, 1.7 μm), the chromatographic separation was performed with a mobile phase containing 0.1% formic acid in water (solvent A) and acetonitrile (solvent B). The specific conditions for the optimization of the elution gradient were as reported in the previous study (Yoo, Kim, et al., 2022). The acquired raw data were subsequently subjected to processing using the Progenesis QI software (Nonlinear Dynamics, Newcastle upon Tyne, UK).

### 2.3 Animals

All animal experiments adhered to the approved guidelines set by the Institutional Animal Care and Use Committee (IACUC) of the Korea Food Research Institute (KFRI‐M‐19032). Five‐week‐old male C57BL/6 mice were kept under standardized conditions, maintaining a controlled environment with consistent ambient temperature (21–25°C) and humidity (50%–60%). A 12 h light/12 h dark cycle was maintained, and the mice had ad libitum access to both food and water. Following a 1‐week acclimation period, the mice were exposed to an 8‐week exposure to a high‐fat diet (HFD; Research Diets, New Brunswick, NJ, USA) containing 45% of calories from fat to induce obesity. Subsequently, the HFD‐induced obese mice were divided into two groups and received either a continued HFD with oral administration of distilled water (HFD, *n* = 8) or GC (100 mg/kg dissolved in distilled water, GC, *n* = 8) for an additional 8‐week period. An age‐matched control group of mice was kept on a chow diet (chow, *n* = 8) for the same duration. The composition of the experimental diets is shown in Supplementary Table S1. During the experimental period, weekly evaluations of body weight and food intake were carried out. At the end of the 8‐week intervention, the mice were euthanized, and blood samples were collected, followed by the immediate excision and measurement of adipose and muscle tissues. Serum levels of tumor necrosis factor alpha (TNFα), monocyte chemoattractant protein 1 (MCP1), and interleukin 1‐beta (IL‐1β) were measured using commercially available ELISA kits from BioLegend (San Diego, CA, USA) and Abcam (Cambridge, MA, USA). Quadriceps muscle triglyceride (TG) levels were quantified using commercial kits (Abcam).

### 2.4 Treadmill exercise and grip strength assessments

Prior to the running test, all mice underwent a treadmill acclimation process using a treadmill (Ugo Basile, Gemonio, ltaly) for a duration of 2 days. Detailed experimental conditions were implemented with reference to the previous study (Yoo, Ahn, et al., 2022). Grip strength measurements were obtained using a grip strength meter (model GS3, Bioseb, Vitrolles, France). The recorded values were standardized based on body weight, and the average value was determined, with exclusion of the maximum and minimum values.

### 2.5 Hematoxylin and eosin (H&E) staining

Following dissection, the gastrocnemius muscle was immediately fixed in 4% formaldehyde and subsequently embedded in paraffin to obtain 4 μm thick sections. H&E staining was then carried out on the gastrocnemius sections. Microscopic images were obtained using the Olympus BX51 microscope, and the cross‐sectional area (CSA) was measured using IMT iSolution DT 9.2 software.

### 2.6 Cell culture

C2C12 myoblast cells (ATCC, Manassas, VA, USA) were cultured in DMEM containing 10% fetal bovine serum, 100 U/mL penicillin, and 100 μg/mL streptomycin. The cells were maintained in a humidified environment at 37°C with 5% CO_2_. To induce differentiation, C2C12 cells were seeded at a density of 2 × 10^5^ cells per well in 6‐well plates. Two days later, approximately 100% confluent C2C12 cells were transferred to DMEM supplemented with 2% horse serum, 100 U/mL penicillin, and 100 μg/mL streptomycin. On the initiation of differentiation (day 0), the cells were subjected to treatment either with or without the SIRT1‐specific inhibitor, 10 μM EX‐527 (Sigma‐Aldrich, St. Louis, MO, USA) for a period of 1 day, followed by treatment with GC and/or EX‐527 for an additional 2 days.

### 2.7 Western blot analysis

Protein extraction from the quadriceps muscle and C2C12 cells was performed using RIPA buffer. The protein concentration of 20 μg in the supernatant was determined using a Pierce BCA protein assay kit (Thermo Scientific, Rockford, IL, USA) with bovine serum albumin as the standard, following established procedures (He, 2011). Next, total protein was separated by SDS‐PAGE and transferred onto polyvinylidene difluoride membranes (Bio‐Rad, Hercules, CA, USA). The membranes were subjected to blocking using 5% skimmed milk and 0.1% Tween 20 (Junsei, Tokyo, Japan) in Tris‐buffered saline for a duration of 1 h at 25°C. Primary antibodies were allowed to incubate overnight at 4°C with the membranes, followed by thorough washing and subsequent 1 h incubation at 25°C with a horseradish peroxidase‐conjugated secondary antibody. Immunodetection was performed utilizing an ECL detection reagent (Bio‐Rad), and the subsequent quantification of band density was carried out through analysis with ImageJ software (National Institutes of Health, Bethesda, MD, USA).

### 2.8 Quantitative real‐time PCR

The extraction of total RNA from the quadriceps muscle was achieved through the utilization of the Qiagen RNeasy Fibrous Tissue Mini Kit (Qiagen Inc., Hilden, Germany). Following RNA extraction (30 ng/μL), the synthesis of cDNA was performed using the ReverTra Ace® quantitative RT‐PCR master kit (Toyobo Co., Ltd., Osaka, Japan). Quantitative PCR was conducted using SYBR Green real‐time PCR Master Mix (Toyobo Co., Ltd.) along with the ViiA7 PCR system (Applied Biosystems, Foster City, CA, USA). The amplification reaction, comprising 20 μL, included cDNA as the template. The amplification protocol commenced with an initial step at 95°C for 5 min, succeeded by 40 amplification cycles of 95°C for 5 s, 55°C for 10 s, and 72°C for 15 sec.

### 2.9 Statistical analysis

The data are reported as the mean ± SEM. Statistical analyses were performed using GraphPad Prism software, version 8.3.1 (San Diego, CA, USA). To assess differences between two groups, an unpaired *t*‐test was employed. For comparisons involving more than two groups, one‐way analysis of variance (ANOVA) was utilized, followed by Dunnett's multiple comparison test.

## 3 RESULTS

### 3.1 GC decreased ectopic fat accumulation in skeletal muscle and increased skeletal muscle weight, size, grip strength, and treadmill running in HFD‐fed mice

**FIGURE 1:**
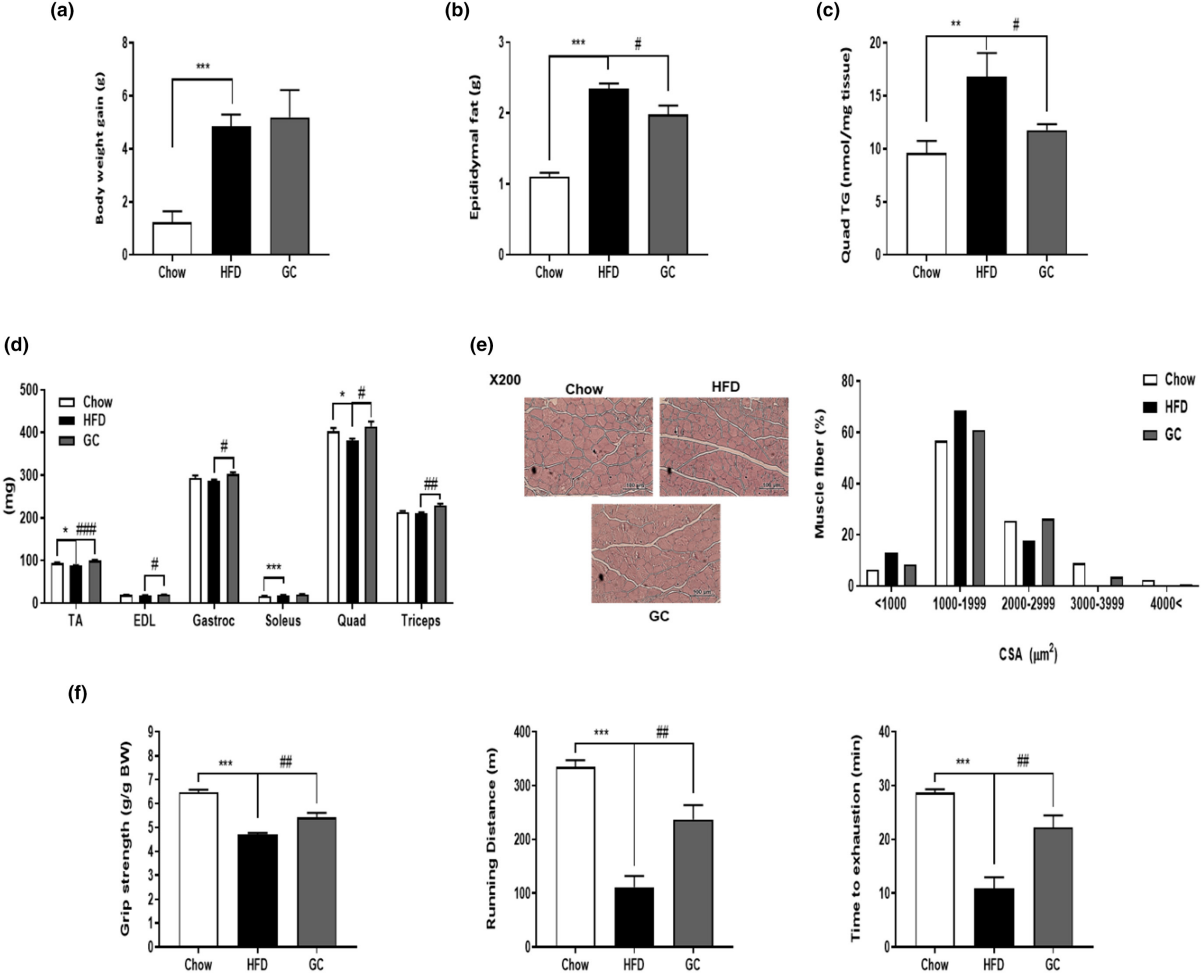
Effect of GC on skeletal muscle size and muscle performance in C57BL/6 mice. (a) Body weight gain, (b) epididymal fat weight of the mice, (c) TG levels in the quadriceps muscle, (d) skeletal muscle weight, (e) a representative image displaying H&E staining of myofiber cross‐sections in the gastrocnemius muscle, and (f) the measured grip strength (g/g BW) and running distance (m and min) as indicators of muscle function. Results are expressed as mean ± SEM. *p < .05, **p < .01, ***p < .001 versus the Chow group. #*p* < .05, ##*p* < .01, ###*p* < .001 versus the HFD‐fed group.

To evaluate the impact of GC on obesity‐associated muscle wasting, we initially recorded body weight and quantified epididymal fat mass. In addition, the TG content within skeletal muscle was measured. *HFD‐fed mice showed elevated* body (*p* < .001) and epididymal fat (*p* < .001) weights in comparison to those fed a chow diet. In the GC group, there was no change in body weight (Figure 1a); however, it was observed that the GC group had decreased epididymal fat weights (*p* < .05) (Figure 1b) compared with those of the HFD group. Moreover, the GC group exhibited a notable decrease in HFD‐induced TG accumulation within the skeletal muscle (*p* < .05) (Figure 1c). In comparison to the chow‐fed group, the HFD group exhibited decreased muscle weight (TA, *p* < .05, Quad, *p* < .05), while the GC group showed elevated skeletal muscle weight relative to the HFD‐fed mice (TA, *p* < .001, EDL, *p* < .05, Gastroc, *p* < .05, Quad, *p* < .05, Triceps, *p* < .01) (Figure 1d). Furthermore, the HFD‐feeding resulted in a reduction in the CSA of skeletal muscle fibers compared to that observed in chow‐fed mice, whereas GC administration led to an augmentation of muscle fiber size relative to the HFD group (Figure 1e). These findings suggest that GC mitigated obesity‐induced fat accumulation in obese mice, leading to an increase in both skeletal muscle weight and size. Furthermore, grip strength and treadmill running were assessed to investigate muscle function. The HFD group showed decreased grip strength (*p* < .001), running distance (*p* < .001), and time to exhaustion (*p* < .001) compared with those of the chow group. However, GC significantly enhanced grip strength (*p* < .01), running distance (*p* < .01), and time to exhaustion (*p* < .01) in a noteworthy manner (Figure 1f). These findings suggest that GC administration led to improved skeletal muscle performance, as evidenced by enhanced grip strength and treadmill running capacity in mice.

### 3.2 GC ameliorated inflammation and skeletal muscle atrophy induced by obesity in mice

**FIGURE 2:**
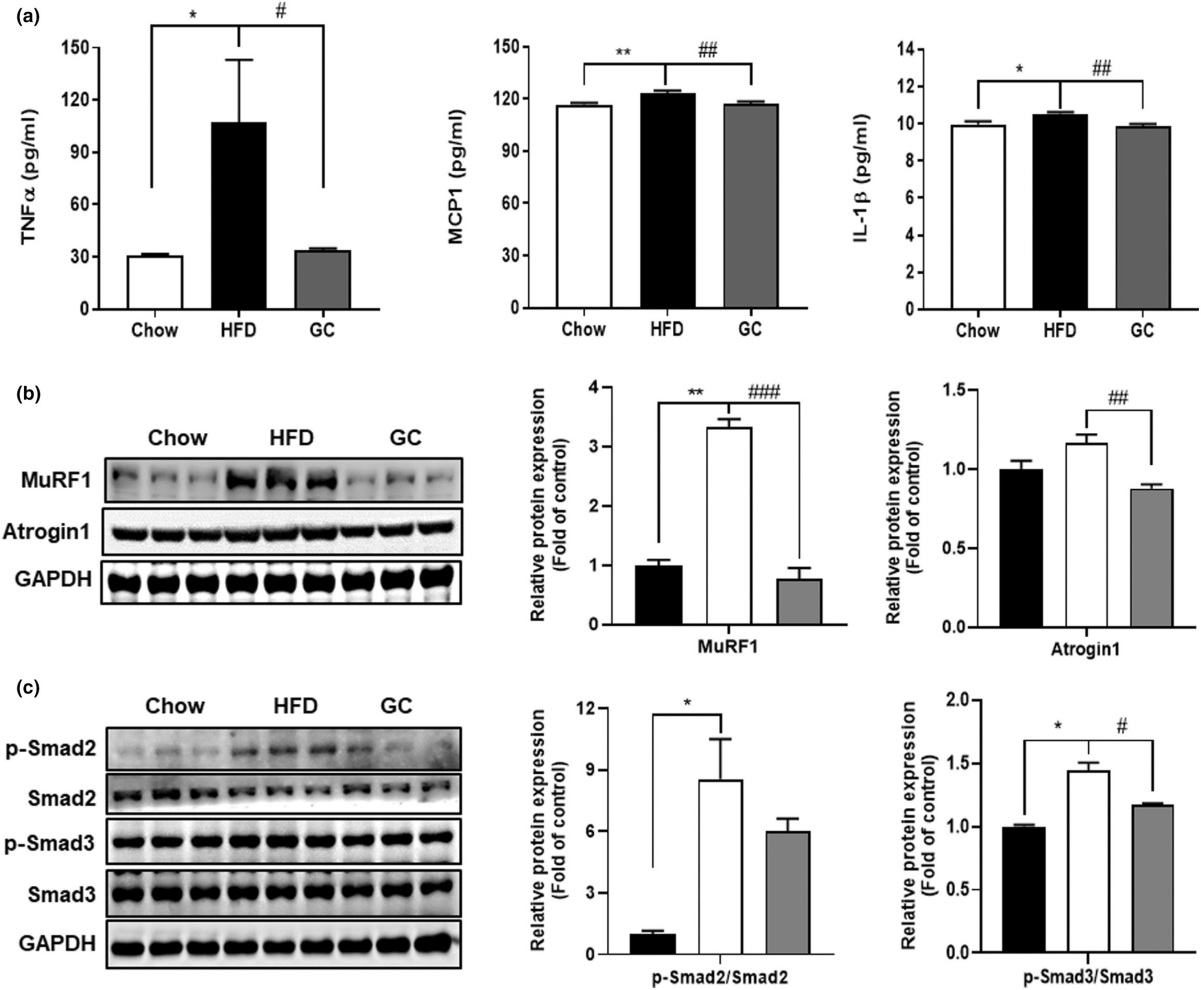
Effect of GC on inflammation and skeletal muscle atrophy induced by obesity in HFD‐fed obese mice. (a) The serum levels of inflammatory cytokines, (b) the protein expression of MuRF1, Atrogin1, and (c) p‐Smad2, Smad2, p‐Smad3, Smad3, and GAPDH in quadriceps muscle as determined by western blotting. Results are expressed as mean ± SEM. *p < .05, **p < .01 versus the Chow group. #*p* < .05, *##p* < .01, ###*p* < .001 versus the HFD‐fed group.

HFD‐feeding increased circulating pro‐inflammatory cytokines, including TNF⍺ (*p* < .05), MCP1 (*p* < .01), and IL‐1β (*p* < .05). However, GC‐fed mice showed significantly decreased serum TNF⍺ (*p* < .05), MCP1 (*p* < .01), and IL‐1β (*p* < .01) levels in comparison to those fed an HFD (Figure 2a). In addition, HFD‐feeding increased skeletal muscle atrophy, resulting in increased protein levels of muscle RING‐finger protein 1 (MuRF1) (*p* < .01) and muscle‐specific ubiquitin E3‐ligases, F‐box protein (MAFbx/Atrogin1), which are skeletal muscle atrophy markers, compared with chow‐fed mice. GC administration led to a significant reduction in the protein levels of MuRF1 (*p* < .001) and Atrogin1 (*p* < .01) (Figure 2b). Phosphorylation of smad2/3 in skeletal muscle mediates the activation of skeletal muscle atrophy through the ubiquitin‐proteasome system (UPS) (Sartori et al., 2014). We observed increased protein levels of phosphorylated Smad2 (*p* < .05) and Smad3 (*p* < .05) in HFD‐fed mice. However, in the GC group, phosphor‐Smad2 and Smad3 (*p* < .05) expression decreased while the total Smad2 and Smad3 protein expression remained unaffected compared to the HFD group (Figure 2c). Taken together, these findings suggest that GC ameliorates obesity‐induced inflammation and skeletal muscle atrophy through its regulatory effect on Smad 2/3 in mice.

### 3.3 GC increased myosin heavy chain (MHC) isoform and skeletal muscle protein synthesis in skeletal muscle of mice

**FIGURE 3:**
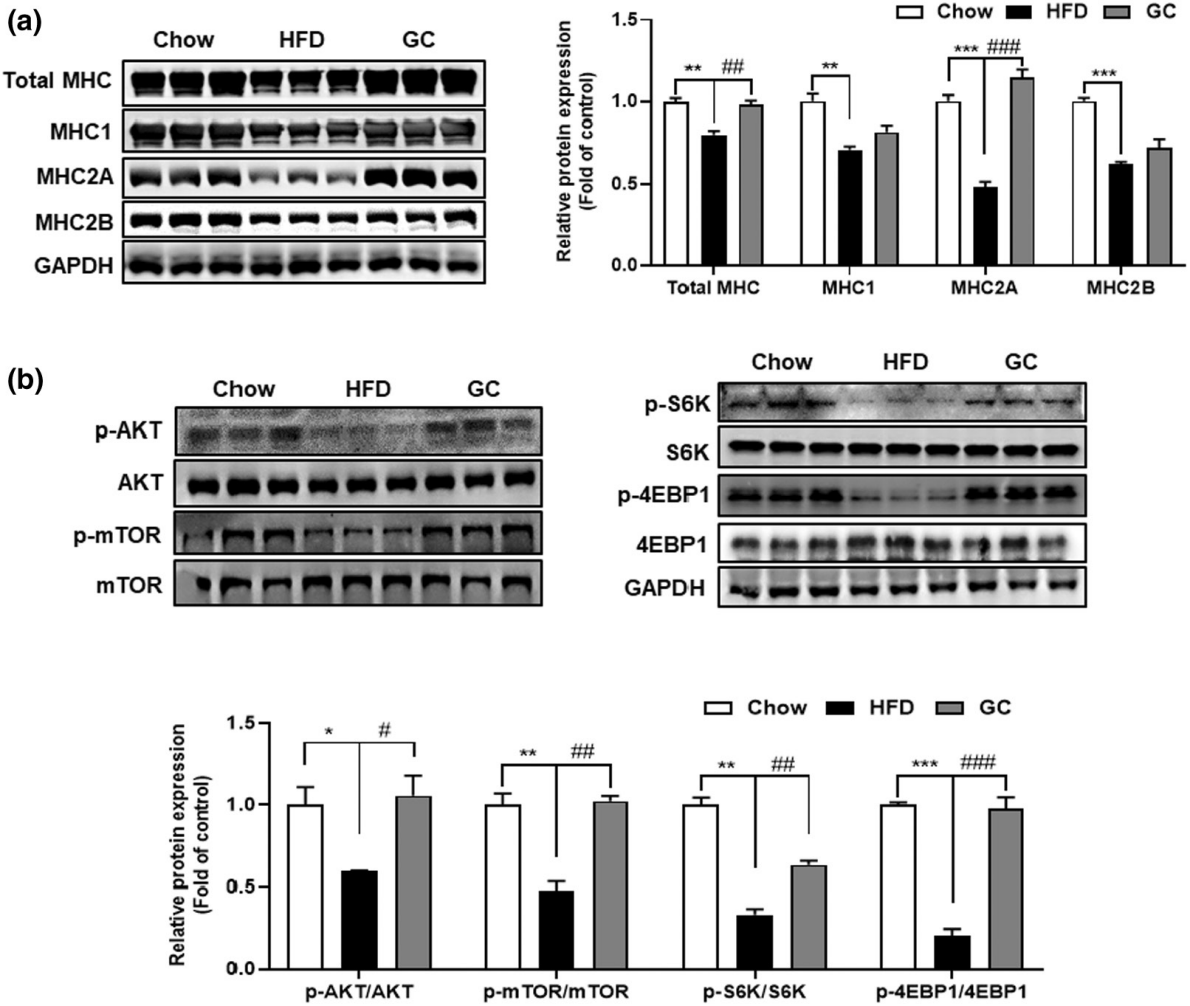
Effect of GC MHC isoform and skeletal muscle protein synthesis in skeletal muscle of mice (a) The protein expression of MHC isoforms, and (b) p‐AKT, AKT, p‐mTOR, mTOR, p‐S6K, S6K, p‐4E‐BP1, 4E‐BP1, and GAPDH in quadriceps muscle was analyzed. Results are expressed as mean ± SEM. *p < .05, **p < .01, ***p < .001 versus the Chow group. #*p <* .05, *##p* < .01, #*##p* < .001 versus the HFD‐fed group.

To investigate the impact of GC on muscle fiber type, we quantified the protein levels of MHC isoforms in the skeletal muscle. HFD‐fed mice reduced total MHC (*p* < .01), MHC1 (*p* < .01), 2A (*p* < .001), and 2B (*p* < .001) protein levels. However, administration of GC resulted in an elevation of total MHC (*p* < .01), MHC1, 2A (*p* < .001), and 2B protein expression in skeletal muscle relative to the HFD group (Figure 3a). In addition, activated Akt phosphorylates and activates mammalian target of rapamycin (mTOR), consequently affecting p70 S6 kinase (S6K) and eIF4E binding protein 1 (4EBP1), which are involved in protein synthesis (Lipina & Hundal, 2017). HFD‐feeding reduced the protein levels of phospho‐AKT (*p* < .05), mTOR (*p* < .01), S6K (*p* < .01), and 4‐EBP1 (*p* < .001) in skeletal muscle in comparison to chow‐fed mice. However, GC increased the expression of phospho‐AKT (*p* < .05), mTOR (*p* < .01), S6K (*p* < .01), and 4EBP1 (*p* < .001) proteins without affecting total AKT, mTOR, S6K, and 4EBP1 protein expression compared with that in the HFD group (Figure 3b). These findings indicate that GC enhances MHC isoforms and promotes skeletal muscle hypertrophy through activation of the AKT/mTOR pathway.

### 3.4 GC ameliorated mitochondrial biogenesis and function via activation of SIRT1/PGC1α, in vivo and in vitro

**FIGURE 4:**
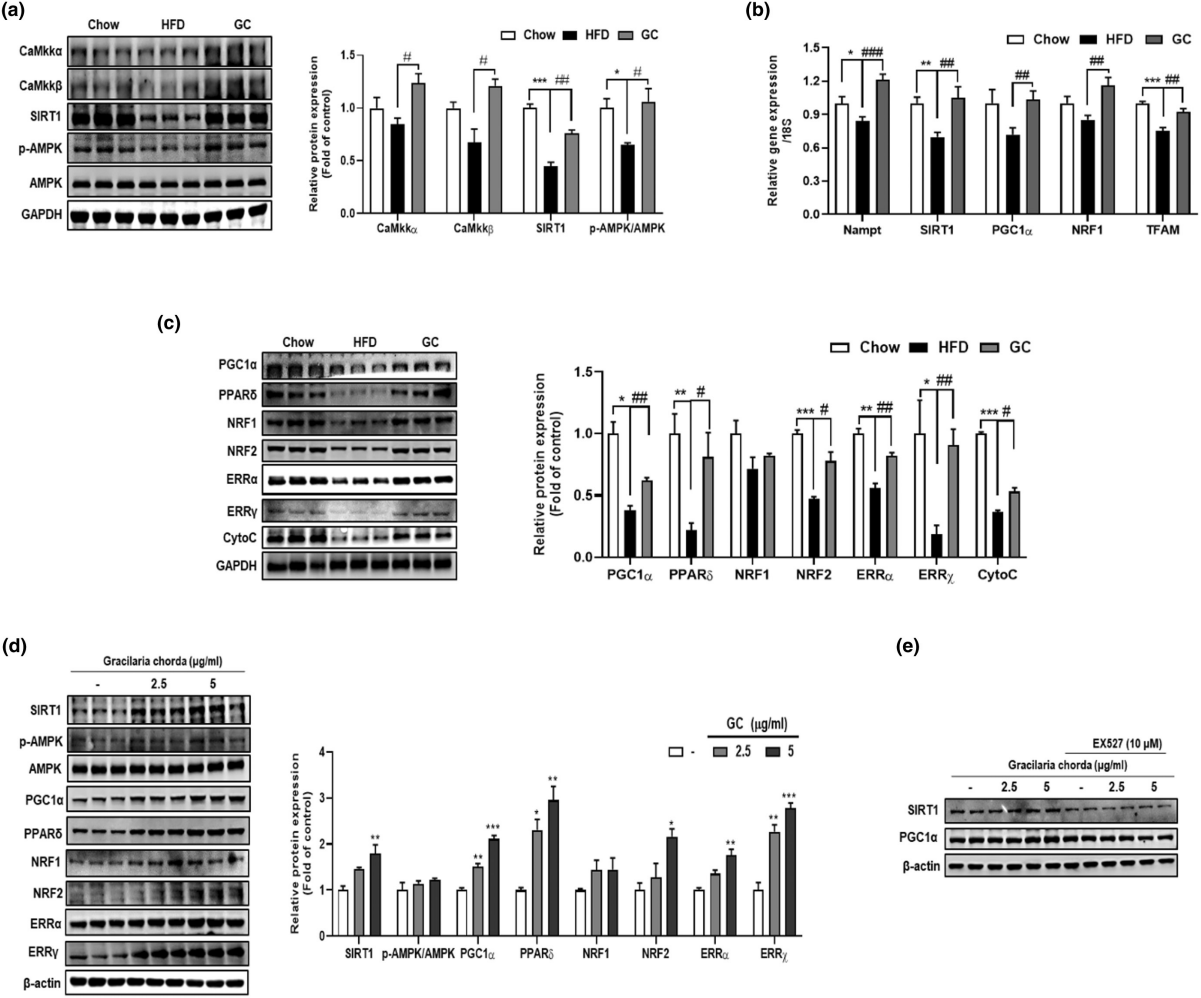
Effect of GC on mitochondrial biogenesis and function through activation of SIRT1/PGC1α in vivo and in vitro. (a) The protein levels of CaMkkα, β, SIRT1, p‐AMPK, AMPK, and GAPDH in the quadriceps muscle were assessed using western blotting. (b) qRT‐PCR was employed to quantify the mRNA expression levels of Nampt, SIRT1, PGC1α, NRF1, and TFAM in the quadriceps muscle. (c) Western blotting was utilized to measure the protein levels of PGC1α, PPARδ, NRF1, NRF2, ERRα, γ, CytoC, and GAPDH in the quadriceps muscle. (d) Western blotting was performed to determine the protein levels of SIRT1, p‐AMPK, AMPK, PGC1α, PPARδ, NRF1, NRF2, ERRα, γ, and β‐Actin in C2C12 cells. (e) Western blotting was used to examine the protein levels of SIRT1 and PGC1α in C2C12 cells treated with GC and/or EX527. Results are expressed as mean ± SEM. *p < .05, **p < .01, ***p < .001 versus the Chow group or the control C2C12 cells. *#p* < .05, *##p* < .01, *###p* < .001 versus the HFD‐fed group.

To examine the impact of GC on mitochondrial biogenesis and function in obese mice, we evaluated the expression of PGC1α, a central regulator of mitochondrial biogenesis and respiratory activity, as well as proteins under the control of PGC1α. Activation of Ca^2+^/calmodulin‐dependent protein kinase (CaMkk) α, β, SIRT1, and AMPK increases PGC1α expression in skeletal muscles (Iwabu et al., 2010). As depicted in Figure 4a, the HFD group exhibited diminished protein expression levels of CaMkkα, CaMkβ, SIRT1 (*p* < .001), and phospho‐AMPK (*p* < .05) in comparison to the chow‐fed mice. However, GC significantly increased CaMkkα (*p* < .05), CaMkβ (*p* < .05), SIRT1 (*p* < .01), and phospho‐AMPK (*p* < .05) protein levels in skeletal muscle compared to those in the HFD group. Furthermore, the HFD group demonstrated reduced mRNA expression of nicotinamide phosphoribosyltransferase (Nampt) (*p* < .05), which subsequently regulates SIRT1 activity, and SIRT1 (*p* < .01), when compared to the chow group. However, GC increased the mRNA expression of Nampt (*p* < .001) and SIRT1 (*p* < .01) in HFD‐fed mice (Figure 4b). HFD‐feeding reduced PGC1α mRNA and protein (*p* < .05) expression relative to the chow group. Subsequently, the mRNA and protein expression of mitochondrial biogenesis and function‐related genes including PPARδ (*p* < .01), nuclear respiratory factor 1 (NRF1), nuclear factor erythroid 2‐related factor 2 (NRF2) (*p* < .001), estrogen‐related receptor (ERR)α (*p* < .01), γ (*p* < .05), cytochrome C (CytoC) (*p* < .001), and transcription factor A (TFAM) (*p* < .001) were also decreased. However, GC increased the mRNA expression of PGC1α (*p* < .01), NRF1 (*p* < .01), and TFAM (*p* < .01), and the protein expression of PGC1α (*p* < .01), PPARδ (*p* < .05), NRF2 (p < .05), ERRα (*p* < .01), γ (*p* < .01), and CytoC (*p* < .05) in HFD‐fed mice (Figure 4b,c). To confirm whether GC increased mitochondrial biogenesis and function, we differentiated C2C12 myoblasts for 1 d and then further differentiated them for 2 d in the presence or absence of GC. GC increased the protein levels of SIRT1 (*p* < .01), phospho‐AMPK, PGC1α (*p* < .01 and *p* < .001), PPARδ (*p* < .05 and *p* < .01), NRF1, NRF2 (*p* < .05), ERRα (*p* < .01), and ERRγ (*p* < .01 and *p* < .001) (Figure 4d). To investigate whether GC increases mitochondrial biogenesis and function via SIRT1, we treated C2C12 cells with or without the SIRT1‐specific inhibitor 10 μM EX‐527 on day 0 of differentiation for 1 d, and subsequently with GC and/or EX‐527 for 2 days. In the absence of EX527 treatment, GC led to heightened protein levels of SIRT1 and PGC1α; however, no change was noted in EX527‐treated C2C12 cells (Figure 4e).

### 3.5 Twenty‐three tentative compounds were identified in GC

**FIGURE 5:**
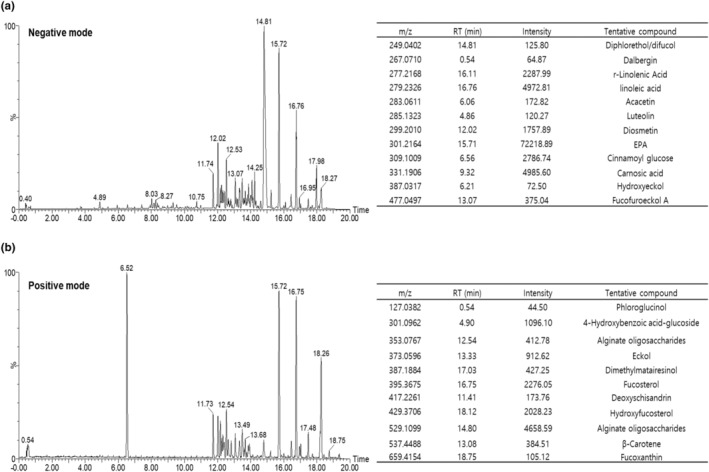
UPLC‐QTOF‐MS/MS analysis of GC extracts. Total ion chromatograms of GC extract in (a) negative mode and (b) positive mode by UPLC‐QTOF‐MS/MS.

The composition of GC was determined by conducting nontargeted analysis using UPLC‐QTOF MS to identify compounds in the GC extract. As shown in Figure 5, 23 tentative compounds are based on accurate mass, retention time, and comparison of MS/MS fragments with standard compounds. In the negative mode, we confirmed the presence of diphlorethol/difucol, dalbergin, r‐linolenic acid, linoleic acid, acacetin, luteolin, diosmetin, eicosapentaenoic acid (EPA), cinnamoyl glucose, carnosic acid, hydroxyeckol, and fucofuroeckol A. In the positive mode, we confirmed the presence of phloroglucinol, 4‐hydroxybenzoic acid‐glucoside, alginate oligosaccharides, eckol, dimethylmatairesinol, fucosterol, deoxyschisandrin, hydroxyfucosterol, alginate oligosaccharides, β‐carotene, and fucoxanthin.

## 4 DISCUSSION

In the present study, GC increased skeletal muscle weight while reducing epididymal fat weight and TG content in skeletal muscle, without a change in body weight in obese mice. Obesity causes increased body fat mass and ectopic fat accumulation in the skeletal muscle, and obesity‐induced increases in pro‐inflammatory cytokines and lipid metabolites lead to an imbalance between muscle hypertrophy and atrophy (Kalinkovich & Livshits, 2017). Therefore, these findings propose that the impact of GC on obesity‐related muscle wasting is due to a decrease in lipid accumulation in skeletal muscle, especially intramuscular lipids. In addition, it has been reported that accumulated muscular lipids induce a muscle lipotoxic effect, reducing muscle strength and power in the elderly (Tumova et al., 2016). As we observed that GC improved skeletal muscle performance, it is expected to be due to a decrease in intramuscular lipids.

In skeletal muscle, the interplay between muscle hypertrophy and atrophy is primarily controlled by the insulin‐like growth factor 1 (IGF‐1)‐AKT–mTOR signaling pathway (Glass, 2010), which exerts a favorable influence on muscle growth, and the myostatin‐Smad2/3 pathway, which serves as a negative regulator (Schiaffino et al., 2013). GC supplementation reduced the increase in pro‐inflammatory cytokines and ameliorated muscle atrophy by regulating Smad2/3, and activated the inhibited AKT signaling, which was suppressed by obesity. It has been reported that obesity suppresses IGF‐1/AKT signaling owing to increased pro‐inflammatory cytokines and toxic lipid metabolites and increases muscle atrophy by allowing phosphorylated Smads to enter the nucleus and regulate MuRF1 and Atrogin1 transcription and active UPS (Glass, 2010; Ji et al., 2022; Mahfouz et al., 2014). Our results suggested that GC improves obesity‐related muscle wasting by activating AKT signaling and suppressing Smad2/3 signaling.

Moreover, GC supplementation significantly increased the expression of SIRT1 and PGC1α, both in vivo and in vitro. SIRT1, an NAD^+^‐dependent protein deacetylase, plays a role in the posttranslational modification of PGC1α through deacetylation. Activated PGC1α, through SIRT1, increases mitochondrial function and biogenesis as crucial genes governing mitochondrial DNA replication, oxidative phosphorylation proteins, and fatty acid oxidation are activated (e.g., PPARδ, NRF1, NRF2, TFAM, ERRα) (Finck & Kelly, 2006; Kelly & Scarpulla, 2004). Next, to investigate whether the increased expression of PGC1α by GC supplementation was mediated by SIRT1, we treated C2C12 cells with GC in the presence of the SIRT1‐specific inhibitor EX‐527. We observed no changes in SIRT1 and PGC1α protein levels in GC‐treated C2C12 cells. These results suggested that SIRT1 mediates the advantageous effects of GC in increasing the expression of genes related to mitochondrial function and biogenesis. In addition, SIRT1 can control muscle atrophy, as SIRT1‐regulated factors contribute to inducing atrophy, and PGC1α can block this process (Sandri et al., 2006) and contribute to muscle growth by increasing AKT (Koltai et al., 2017). This suggested that the activation of SIRT1/PGC1α by GC in skeletal muscle affected mitochondrial function, biogenesis, and skeletal hypertrophy and atrophy.

Activation of PGC1α has been shown to induce muscle fiber‐type transformation in type 1 fibers (Lin et al., 2002). This is because type 1 muscle fibers have a dark red color, a higher mitochondrial density, and more oxidative enzymes than type 2 fibers (Scott et al., 2001). Although the present study did not conduct muscle fiber‐type conversion, it was observed that MHC1 was reduced in HFD‐fed mice. GC increased MHC1 expression. However, it was observed that MHC1, total MHC, MHC2A, and 2B were decreased in HFD‐fed mice. This appears to be due to the reduction in skeletal muscle weight and size, and further studies are required to measure muscle fiber‐type conversion.

We identified 23 tentative compounds in GC. Among them, linoleic acid, luteolin, EPA, deoxyschisandrin, β‐carotene, and fucoxanthin have been reported to prevent muscle atrophy in a muscle atrophy model (Hedya et al., 2019; Lee et al., 2022; Ogawa et al., 2013; Yeon et al., 2020; Yoshikawa et al., 2021) Therefore, several bioactive compounds in GC may exert synergistic effects against obesity‐related muscle wasting. As the bioactive compounds of GC are unknown, future studies are needed to identify the physiologically active compounds of GC through component analysis and to investigate whether these compounds affect obesity‐related muscle wasting.

In conclusion, our findings indicate that GC attenuates obesity‐related muscle wasting by improving mitochondrial function and biogenesis via the activation of SIRT1/PGC1α in the skeletal muscle of mice. Taken together, these findings suggested that GC may be a useful food resource for both preventing and treating obese sarcopenia.

## AUTHOR CONTRIBUTIONS

**Ahyoung Yoo:** Formal analysis (lead); investigation (lead); writing – original draft (lead). **Jiyun Ahn:** Data curation (equal). **Hyo Deok Seo:** Formal analysis (equal); visualization (equal). **Jeong‐Hoon Hahm:** Formal analysis (equal); visualization (equal). **Min Jeong Kim:** Investigation (equal). **Chang Hwa Jung:** Data curation (equal). **Tae Youl Ha:** Conceptualization (lead); supervision (lead); writing – review and editing (lead).

## CONFLICT OF INTEREST STATEMENT

The authors declare that they do not have any conflict of interest.

## ETHICS STATEMENTS

Ethical Review: All animal experiments adhered to the approved guidelines set by the IACUC of the Korea Food Research Institute (KFRI‐M‐19032).

## Supporting information


Table S1.

## Data Availability

The data that support the findings of this study are available from the corresponding author upon reasonable request.

## References

[fsn34157-bib-0001] Armisen, R. (1995). World‐wide use and importance of Gracilaria. Journal of Applied Phycology, 7(3), 231–243.

[fsn34157-bib-0002] Batsis, J. A. , & Villareal, D. T. (2018). Sarcopenic obesity in older adults: Aetiology, epidemiology and treatment strategies. Nature Reviews Endocrinology, 14(9), 513–537. 10.1038/s41574-018-0062-9 PMC6241236 30065268

[fsn34157-bib-0003] Biltz, N. K. , Collins, K. H. , Shen, K. C. , Schwartz, K. , Harris, C. A. , & Meyer, G. A. (2020). Infiltration of intramuscular adipose tissue impairs skeletal muscle contraction. The Journal of Physiology, 598(13), 2669–2683. 32358797 10.1113/JP279595 PMC8767374

[fsn34157-bib-0004] Dang, H. T. , Lee, H. J. , Yoo, E. S. , Shinde, P. B. , Lee, Y. M. , Hong, J. , Kim, D. K. , & Jung, J. H. (2008). Anti‐inflammatory constituents of the red alga Gracilaria verrucosa and their synthetic analogues. Journal of Natural Products, 71(2), 232–240. 18220352 10.1021/np070452q

[fsn34157-bib-0005] Devarshi, P. P. , McNabney, S. M. , & Henagan, T. M. (2017). Skeletal muscle nucleo‐mitochondrial crosstalk in obesity and type 2 diabetes. International Journal of Molecular Sciences, 18(4), 831. 28420087 10.3390/ijms18040831 PMC5412415

[fsn34157-bib-0006] Faten, M. , & Emad, A. S. (2009). Antioxidant activity of extract and semi‐purified fractions of marine red macroalga, Gracilaria verrucosa. Australian Journal of Basic and Applied Sciences, 3(4), 3179–3185.

[fsn34157-bib-0007] Finck, B. N. , & Kelly, D. P. (2006). PGC‐1 coactivators: Inducible regulators of energy metabolism in health and disease. The Journal of Clinical Investigation, 116(3), 615–622. 16511594 10.1172/JCI27794 PMC1386111

[fsn34157-bib-0008] Glass, D. J. (2010). PI3 kinase regulation of skeletal muscle hypertrophy and atrophy. Current Topics in Microbiology and Immunology, 346, 267–278. 20593312 10.1007/82_2010_78

[fsn34157-bib-0009] He, F. (2011). BCA (bicinchoninic acid) protein assay. Bio‐Protocol, 1(5), e44.

[fsn34157-bib-0010] Hedya, S. , Hawila, N. , Abdin, A. , & Maaly, A. E. (2019). Luteolin attenuates dexamethasone‐induced skeletal muscle atrophy in male albino rats. The Medical Journal of Cairo University, 87, 3365–3374.

[fsn34157-bib-0011] Hong, S.‐M. , Cho, H.‐D. , Kim, J.‐H. , Lee, J.‐H. , Song, W.‐S. , Lee, S.‐T. , Lee, M.‐K. , & Seo, K.‐I. (2016). Anti‐proliferative effects of acid extract of Gracilaria verrucosa on primary human prostate cancer cells. Journal of Life Sciences, 26(10), 1130–1136.

[fsn34157-bib-0012] Iwabu, M. , Yamauchi, T. , Okada‐Iwabu, M. , Sato, K. , Nakagawa, T. , Funata, M. , Yamaguchi, M. , Namiki, S. , Nakayama, R. , Tabata, M. , Ogata, H. , Kubota, N. , Takamoto, I. , Hayashi, Y. K. , Yamauchi, N. , Waki, H. , Fukayama, M. , Nishino, I. , Tokuyama, K. , … Tabata, M. (2010). Adiponectin and AdipoR1 regulate PGC‐1α and mitochondria by Ca 2+ and AMPK/SIRT1. Nature, 464(7293), 1313–1319. 20357764 10.1038/nature08991

[fsn34157-bib-0013] Ji, Y. , Li, M. , Chang, M. , Liu, R. , Qiu, J. , Wang, K. , Deng, C. , Shen, Y. , Zhu, J. , Wang, W. , Xu, L. , & Wang, W. (2022). Inflammation: Roles in skeletal muscle atrophy. Antioxidants, 11(9), 1686. 36139760 10.3390/antiox11091686 PMC9495679

[fsn34157-bib-0014] Kalinkovich, A. , & Livshits, G. (2017). Sarcopenic obesity or obese sarcopenia: A cross talk between age‐associated adipose tissue and skeletal muscle inflammation as a main mechanism of the pathogenesis. Ageing Research Reviews, 35, 200–221. 27702700 10.1016/j.arr.2016.09.008

[fsn34157-bib-0015] Kelly, D. P. , & Scarpulla, R. C. (2004). Transcriptional regulatory circuits controlling mitochondrial biogenesis and function. Genes & Development, 18(4), 357–368. 15004004 10.1101/gad.1177604

[fsn34157-bib-0016] Koltai, E. , Bori, Z. , Chabert, C. , Dubouchaud, H. , Naito, H. , Machida, S. , Davies, K. J. , Murlasits, Z. , Fry, A. C. , Boldogh, I. , & Boldogh, I. (2017). SIRT1 may play a crucial role in overload‐induced hypertrophy of skeletal muscle. The Journal of Physiology, 595(11), 3361–3376. 28251652 10.1113/JP273774 PMC5451718

[fsn34157-bib-0017] Kuswari, M. , Nurkolis, F. , Mayulu, N. , Ibrahim, F. M. , Taslim, N. A. , Wewengkang, D. S. , Bahar, M. R. , Sabrina, N. , Arifin, G. R. , Mantik, K. E. K. , Bahar, M. R. , Rifqiyati, N. , Rompies, R. , & Augusta, P. S. (2021). Sea grapes extract improves blood glucose, total cholesterol, and PGC‐1α in rats fed on cholesterol‐and fat‐enriched diet. F1000Research, 10, 718. 35136575 10.12688/f1000research.54952.1 PMC8804902

[fsn34157-bib-0018] Lee, M.‐H. , Lee, J.‐H. , Kim, W.‐J. , Kim, S. H. , Kim, S.‐Y. , Kim, H. S. , & Kim, T.‐J. (2022). Linoleic acid attenuates denervation‐induced skeletal muscle atrophy in mice through regulation of reactive oxygen species‐dependent signaling. International Journal of Molecular Sciences, 23(9), 4778. 35563168 10.3390/ijms23094778 PMC9105847

[fsn34157-bib-0019] Lin, J. , Wu, H. , Tarr, P. T. , Zhang, C.‐Y. , Wu, Z. , Boss, O. , Michael, L. F. , Puigserver, P. , Isotani, E. , Olson, E. N. , Lowell, B. B. , Bassel‐Duby, R. , & Olson, E. N. (2002). Transcriptional co‐activator PGC‐1α drives the formation of slow‐twitch muscle fibres. Nature, 418(6899), 797–801. 12181572 10.1038/nature00904

[fsn34157-bib-0020] Lipina, C. , & Hundal, H. S. (2017). Lipid modulation of skeletal muscle mass and function. Journal of Cachexia, Sarcopenia and Muscle, 8(2), 190–201. 27897400 10.1002/jcsm.12144 PMC5377414

[fsn34157-bib-0021] Mahfouz, R. , Khoury, R. , Blachnio‐Zabielska, A. , Turban, S. , Loiseau, N. , Lipina, C. , Stretton, C. , Bourron, O. , Ferré, P. , Foufelle, F. , Hundal, H. S. , & Foufelle, F. (2014). Characterising the inhibitory actions of ceramide upon insulin signaling in different skeletal muscle cell models: A mechanistic insight. PLoS ONE, 9(7), e101865. 10.1371/journal.pone.0101865 25058613 PMC4109934

[fsn34157-bib-0022] Manoppo, J. I. C. , Nurkolis, F. , Pramono, A. , Ardiaria, M. , Murbawani, E. A. , Yusuf, M. , Qhabibi, F. R. , Yusuf, V. M. , Amar, N. , Karim, M. R. A. , Subali, A. D. , Natanael, H. , Rompies, R. , Halim, R. F. , Bolang, A. S. L. , Joey, G. , Novianto, C. A. , & Karim, M. R. A. (2022). Amelioration of obesity‐related metabolic disorders via supplementation of Caulerpa lentillifera in rats fed with a high‐fat and high‐cholesterol diet. Frontiers in Nutrition, 9, 2124. 10.3389/fnut.2022.1010867 PMC9521187 36185651

[fsn34157-bib-0023] Mohibbullah, M. , Abdul Hannan, M. , Park, I.‐S. , Moon, I. S. , & Hong, Y.‐K. (2016). The edible red seaweed *Gracilariopsis chorda* promotes axodendritic architectural complexity in hippocampal neurons. Journal of Medicinal Food, 19(7), 638–644. 27331292 10.1089/jmf.2016.3694

[fsn34157-bib-0024] Mohibbullah, M. , Choi, J.‐S. , Bhuiyan, M. M. H. , Haque, M. N. , Rahman, M. K. , Moon, I. S. , & Hong, Y.‐K. (2018). The red alga *Gracilariopsis chorda* and its active constituent arachidonic acid promote spine dynamics via dendritic filopodia and potentiate functional synaptic plasticity in hippocampal neurons. Journal of Medicinal Food, 21(5), 481–488. 29498567 10.1089/jmf.2017.4026

[fsn34157-bib-0025] Mohibbullah, M. , Hannan, M. A. , Choi, J.‐Y. , Bhuiyan, M. M. H. , Hong, Y.‐K. , Choi, J.‐S. , Choi, I. S. , & Moon, I. S. (2015). The edible marine alga *Gracilariopsis chorda* alleviates hypoxia/reoxygenation‐induced oxidative stress in cultured hippocampal neurons. Journal of Medicinal Food, 18(9), 960–971. 26106876 10.1089/jmf.2014.3369 PMC4580144

[fsn34157-bib-0026] Nurkolis, F. , Taslim, N. A. , Subali, D. , Kurniawan, R. , Hardinsyah, H. , Gunawan, W. B. , Kusuma, R. J. , Yusuf, V. M. , Pramono, A. , Kang, S. , Mayulu, N. , Syauki, A. Y. , Tallei, T. E. , Tsopmo, A. , & Kang, S. (2023). Dietary supplementation of *Caulerpa* racemosa ameliorates Cardiometabolic syndrome via regulation of PRMT‐1/DDAH/ADMA pathway and gut microbiome in mice. Nutrients, 15(4), 909. 36839268 10.3390/nu15040909 PMC9959712

[fsn34157-bib-0027] Ogawa, M. , Kariya, Y. , Kitakaze, T. , Yamaji, R. , Harada, N. , Sakamoto, T. , Hosotani, K. , Nakano, Y. , & Inui, H. (2013). The preventive effect of β‐carotene on denervation‐induced soleus muscle atrophy in mice. British Journal of Nutrition, 109(8), 1349–1358. 23046823 10.1017/S0007114512003297

[fsn34157-bib-0028] Park, S. Y. , Cho, W. , Abd El‐Aty, A. , Hacimuftuoglu, A. , Jeong, J. H. , & Jung, T. W. (2022). Valdecoxib attenuates lipid‐induced hepatic steatosis through autophagy‐mediated suppression of endoplasmic reticulum stress. Biochemical Pharmacology, 199, 115022. 35358477 10.1016/j.bcp.2022.115022

[fsn34157-bib-0029] Poggiogalle, E. , Rossignon, F. , Carayon, A. , Capel, F. , Rigaudière, J.‐P. , de Saint Vincent, S. , Le‐Bacquer, O. , Salles, J. , Giraudet, C. , Patrac, V. , Lebecque, P. , Walrand, S. , Boirie, Y. , Martin, V. , & Guillet, C. (2022). Deleterious effect of high‐fat diet on skeletal muscle performance is prevented by high‐protein intake in adult rats but not in old rats. Frontiers in Physiology, 12, 749049. 35111075 10.3389/fphys.2021.749049 PMC8801536

[fsn34157-bib-0030] Rubio‐Ruiz, M. E. , Guarner‐Lans, V. , Pérez‐Torres, I. , & Soto, M. E. (2019). Mechanisms underlying metabolic syndrome‐related sarcopenia and possible therapeutic measures. International Journal of Molecular Sciences, 20(3), 647. 30717377 10.3390/ijms20030647 PMC6387003

[fsn34157-bib-0031] Ryan, A. S. , & Li, G. (2021). Skeletal muscle myostatin gene expression and sarcopenia in overweight and obese middle‐aged and older adults. JCSM Clinical Reports, 6(4), 137–142. 35311023 10.1002/crt2.43 PMC8932637

[fsn34157-bib-0032] Sandri, M. , Lin, J. , Handschin, C. , Yang, W. , Arany, Z. P. , Lecker, S. H. , Goldberg, A. L. , & Spiegelman, B. M. (2006). PGC‐1α protects skeletal muscle from atrophy by suppressing FoxO_3_ action and atrophy‐specific gene transcription. Proceedings of the National Academy of Sciences, 103(44), 16260–16265. 10.1073/pnas.0607795103 PMC1637570 17053067

[fsn34157-bib-0033] Sartori, R. , Gregorevic, P. , & Sandri, M. (2014). TGFβ and BMP signaling in skeletal muscle: Potential significance for muscle‐related disease. Trends in Endocrinology and Metabolism, 25(9), 464–471. 25042839 10.1016/j.tem.2014.06.002

[fsn34157-bib-0034] Schiaffino, S. , Dyar, K. A. , Ciciliot, S. , Blaauw, B. , & Sandri, M. (2013). Mechanisms regulating skeletal muscle growth and atrophy. The FEBS Journal, 280(17), 4294–4314. 23517348 10.1111/febs.12253

[fsn34157-bib-0035] Scott, W. , Stevens, J. , & Binder–Macleod, S. A. (2001). Human skeletal muscle fiber type classifications. Physical Therapy, 81(11), 1810–1816. 11694174

[fsn34157-bib-0036] Tumova, J. , Andel, M. , & Trnka, J. (2016). Excess of free fatty acids as a cause of metabolic dysfunction in skeletal muscle. Physiological Research, 65(2), 193–207. 26447514 10.33549/physiolres.932993

[fsn34157-bib-0037] Woo, M. S. , Choi, H. S. , Lee, O. H. , & Lee, B. Y. (2013). The edible red alga, Gracilaria verrucosa, inhibits lipid accumulation and ROS production, but improves glucose uptake in 3T3‐L1 cells. Phytotherapy Research, 27(7), 1102–1105. 22991308 10.1002/ptr.4813

[fsn34157-bib-0038] Yeon, M. , Choi, H. , & Jun, H.‐S. (2020). Preventive effects of Schisandrin a, a bioactive component of Schisandra chinensis, on dexamethasone‐induced muscle atrophy. Nutrients, 12(5), 1255. 32354126 10.3390/nu12051255 PMC7282012

[fsn34157-bib-0039] Yoo, A. , Ahn, J. , Kim, M. J. , Seo, H.‐D. , Hahm, J.‐H. , Jung, C. H. , & Ha, T. Y. (2022). Fruit of Schisandra chinensis and its bioactive component schizandrin B ameliorate obesity‐induced skeletal muscle atrophy. Food Research International, 157, 111439. 35761679 10.1016/j.foodres.2022.111439

[fsn34157-bib-0040] Yoo, A. , Kim, M. J. , Ahn, J. , Jung, C. H. , Seo, H. D. , Ly, S. Y. , & Ha, T. Y. J. F. (2022). Fuzhuan brick tea extract prevents diet‐induced obesity via stimulation of fat browning in mice. Food Chemistry, 377, 132006. 34999463 10.1016/j.foodchem.2021.132006

[fsn34157-bib-0041] Yoshikawa, M. , Hosokawa, M. , Miyashita, K. , Nishino, H. , & Hashimoto, T. (2021). Effects of fucoxanthin on the inhibition of dexamethasone‐induced skeletal muscle loss in mice. Nutrients, 13(4), 1079. 33810214 10.3390/nu13041079 PMC8066636

[fsn34157-bib-0042] Zhu, S. , Tian, Z. , Torigoe, D. , Zhao, J. , Xie, P. , Sugizaki, T. , Sato, M. , Horiguchi, H. , Terada, K. , Kadomatsu, T. , Miyata, K. , & Kadomatsu, T. (2019). Aging‐and obesity‐related peri‐muscular adipose tissue accelerates muscle atrophy. PLoS ONE, 14(8), e0221366. 31442231 10.1371/journal.pone.0221366 PMC6707561

