# Supplementary material for: Gracilaria chorda attenuates obesity‐related muscle wasting through activation of SIRT1/PGC1α in skeletal muscle of mice
Source: Food Sci Nutr. 2024 Apr 4;12(7):5077–86. doi: 10.1002/fsn3.4157 (PMC11266886; doi:10.1002/fsn3.4157)
Supplement: Supplementary file 1 — Table S1. [file FSN3-12-5077-s001.docx]

**Supplementary Table 1. Composition and energy content of chow and high-fat diet**

| Content (Kcal%) | Diets | |
| --- | --- | --- |
|  | Chow | High-fat diet |
| Fat %  Protein %  Carbohydrate %  Energy (Kcal/g)  Protein ingredients  Fat ingredients  Carbohydrate ingredients | 18  24  58  3.1  -  -  - | 45  20  35  4.73  Casein, L-Cystine  Soybean oil, Lard  Corn Starch, Maltodextrin, Sucrose |
